# Distinctive diets of eutherian predators in Australia

**DOI:** 10.1098/rsos.220792

**Published:** 2022-10-12

**Authors:** Patricia A. Fleming, Alyson M. Stobo-Wilson, Heather M. Crawford, Stuart J. Dawson, Chris R. Dickman, Tim S. Doherty, Peter J. S. Fleming, Thomas M. Newsome, Russell Palmer, Jim A. Thompson, John C. Z. Woinarski

**Affiliations:** ^1^ Centre for Terrestrial Ecosystem Science and Sustainability, Harry Butler Institute, Murdoch University, 90 South Street, Murdoch, Western Australia 6150, Australia; ^2^ NESP Threatened Species Recovery Hub, Charles Darwin University, Casuarina, Northern Territory 0909, Australia; ^3^ CSIRO Land and Water, PMB 44, Winnellie, Northern Territory 0822, Australia; ^4^ Department of Primary Industries and Regional Development, 3 Baron-Hay Court, South Perth, Western Australia 6151, Australia; ^5^ Desert Ecology Research Group, School of Life and Environmental Sciences, The University of Sydney, Heydon-Laurence Building A08, Camperdown, New South Wales 2006, Australia; ^6^ School of Life and Environmental Sciences, The University of Sydney, Heydon-Laurence Building A08, Camperdown, New South Wales 2006, Australia; ^7^ Vertebrate Pest Research Unit, NSW Department of Primary Industries, Orange Agricultural Institute, 1447 Forest Road, Orange, New South Wales 2800, Australia; ^8^ Ecosystem Management, School of Environmental and Rural Science, University of New England, Armidale, New South Wales 2351, Australia; ^9^ Institute for Agriculture and the Environment, Centre for Sustainable Agricultural Systems, University of Southern Queensland, Toowoomba, Queensland 4350, Australia.; ^10^ Department of Biodiversity, Conservation and Attractions, Locked Bag 104, Bentley Delivery Centre, Western Australia 6983, Australia; ^11^ Queensland Museum Network, PO Box 3300, South Brisbane BC, Queensland 4101, Australia; ^12^ Research Institute for the Environment and Livelihoods, Charles Darwin University, Casuarina, Northern Territory 0909, Australia

**Keywords:** *Canis familiaris*, *Felis catus*, invasive species, niche separation, resource partitioning, *Vulpes vulpes*

## Abstract

Introduction of the domestic cat and red fox has devastated Australian native fauna. We synthesized Australian diet analyses to identify traits of prey species in cat, fox and dingo diets, which prey were more frequent or distinctive to the diet of each predator, and quantified dietary overlap. Nearly half (45%) of all Australian terrestrial mammal, bird and reptile species occurred in the diets of one or more predators. Cat and dingo diets overlapped least (0.64 ± 0.27, *n* = 24 location/time points) and cat diet changed little over 55 years of study. Cats were more likely to have eaten birds, reptiles and small mammals than foxes or dingoes. Dingo diet remained constant over 53 years and constituted the largest mammal, bird and reptile prey species, including more macropods/potoroids, wombats, monotremes and bandicoots/bilbies than cats or foxes. Fox diet had greater overlap with both cats (0.79 ± 0.20, *n* = 37) and dingoes (0.73 ± 0.21, *n* = 42), fewer distinctive items (plant material, possums/gliders) and significant spatial and temporal heterogeneity over 69 years, suggesting the opportunity for prey switching (especially of mammal prey) to mitigate competition. Our study reinforced concerns about mesopredator impacts upon scarce/threatened species and the need to control foxes and cats for fauna conservation. However, extensive dietary overlap and opportunism, as well as low incidence of mesopredators in dingo diets, precluded resolution of the debate about possible dingo suppression of foxes and cats.

## 1. Introduction

Introduced predators are a major cause of animal extinctions globally [1,2]. In Australia, they threaten many mammal, bird and squamate reptile species [3], and are a documented major driver of native mammal declines and extinctions [4,5]. The arrival of eutherian predators, including the dingo (*Canis familiaris* Linnaeus), red fox (*Vulpes vulpes* (Linnaeus); hereafter ‘fox’) and domestic cat (*Felis catus* Linnaeus; hereafter ‘cat’)—and their larger body sizes and different hunting strategies from most recent native marsupial carnivores [6]—has had a significant impact on the Australian fauna, which evolved in relative isolation for 60 Myr. The dingo was likely introduced to mainland Australia by Asian seafarers between 5000 and 3600 years ago [7,8], is a top-order predator and is now often considered a native species (e.g. [9]). The domestic cat, introduced approximately 230 years ago, and the red fox, introduced about 150 years ago, were introduced by Europeans [10], and their geographical ranges have expanded steadily since.

Australian native rodents and smaller marsupials are exceptionally vulnerable to predation by the cat and fox, and to a lesser extent the dingo. The impacts of predation on native prey numbers have been most severe for species of intermediate body sizes—terrestrial and non-volant species in a ‘critical weight range’ between 35 g and 5.5 kg [11–14]. Other native species such as kangaroos (Macropodidae) and wombats (Vombatidae) generally have the advantage of larger body size, although their juveniles are susceptible to predation (e.g. [15]). Other traits such as activity patterns and habitat use (e.g. where and when they forage and take refuge) are also likely to influence predation risk. Understanding predator diets can therefore help reveal which animal species are more likely to be killed and eaten, and where conservation action should be prioritized.

The fox and cat have broadly overlapping geographical ranges with the dingo, although there are marked differences in predator density and habitat use across the continent [16,17]. There is debate in Australia about whether dingoes suppress the abundance of introduced mesopredators (cats and foxes) and thereby decrease the overall predation pressure on prey, especially when resources are shared with the top-order predator [18–20]. The potential mechanisms for mesopredator suppression by dingoes include interference competition (i.e. via aggression towards or killing the subordinate predator) and exploitation competition for common resources (e.g. food—either killed or scavenged; or space/habitat). Because occurrence of a species in a predator's diet does not provide information about population changes of prey or potentially competitive predators, it is impossible to establish interference or exploitation competition from dietary studies. However, they provide knowledge of dietary resource partitioning or potential interguild predation addressing both forms of competition and possible mechanisms for regulating mesopredators and their impacts on vulnerable prey. Additional syntheses of population and behavioural interactions between cats, foxes and dingoes are required to quantify the extent of any suppression of the mesopredators by dingoes.

Reviews of predator diets are influenced by the study location, as ecogeography determines prey availability [21–23]. The timing of a study, both within and between years, also influences its outcomes as thee abundances of invertebrates, amphibians, reptiles, migratory birds and fruit are often highly seasonal. Further, long-term trends or stochastic events also influence the results of dietary studies. For example, the introduction of successive waves of biocontrol to reduce populations of the introduced rabbit (*Oryctolagus cuniculus* (Linnaeus)) in Australia has reduced the availability of rabbits as prey from the plague numbers witnessed in the 1920s [24,25]. There has also been a steady increase in human population and disturbance across urban and agricultural landscapes, a shift away from sheep, *Ovis aries* Linnaeus*,* production since the 1970s in the wake of decreased wool prices, and an increase in cattle, *Bos taurus* Linnaeus*/B. indicus* Linnaeus, numbers in some areas [26]. Broadscale comparison of cat, fox and dingo diets should therefore account for spatial and temporal differences between studies.

This study broadly follows on from a suite of recent studies focusing on analyses of large diet datasets compiled for Australian cats [27–32], foxes [33–35] and dingoes [22]. The current study differs by providing comparative analysis of diet composition of all three species, quantifies dietary overlap between these species and identifies environmental factors that influence dietary overlap. The Australian situation is of considerable conservation importance, as this small complement of eutherian predator species now co-occur across most of the continent. They likely far outstrip the ecological influence of the largest native predatory mammal species (comprising four *Dasyurus* species and the Tasmanian devil *Sarcophilus harrisii* (Boitard)), which all now have smaller geographical ranges and are threatened, and because invasive introduced animals often dominate and suppress native species and grossly modify species composition, structure and function [36–38].

Here, we investigated the relative consumption of different prey taxa by cats, foxes and dingoes from a large number of dietary studies conducted across the Australian continent over *ca* 70 years to identify dietary overlap and potential competition, and quantify spatial and temporal changes in the diets of these predators. The approach we adopted was as follows:

(1) We examined prey species traits that might increase the likelihood that they are consumed by cats, foxes or dingoes. These included body mass (because it influences species composition of cat and fox diets [31,33–35] and is linked with the impacts of these predators on mammals of conservation significance [11–14]), activity patterns (i.e. overlap with the nocturnally active cat and fox or the crepuscular dingo) and habitat use, traits that have been used to predict the potential vulnerability of native fauna to cat, fox and dingo predation [39–41].
(2) We investigated which prey were more likely to appear in the diets of each predator species by their frequency of occurrence (FOO). Understanding predator diets can help reveal which animal species are more likely to be killed and eaten, and therefore where monitoring programmes may be directed to detect declines in prey populations, and where conservation action should be prioritized.
(3) We investigated what makes the diets of each predator distinctive, as well as the spatial and temporal patterns in dietary composition. We used this information to identify the degree to which these species are specialists (i.e. consuming the same diet irrespective of spatial or temporal variability in prey availability) or generalists/opportunists (i.e. plastically altering their diet in response to spatial or temporal variability in prey availability).
(4) We compared dietary overlap for studies that simultaneously compared the diets of two or even all three predators from the same locations (see references listed in electronic supplementary material, table S1) to understand dietary differences given the same prey availability. We used these data to reveal dietary preferences that can reflect hunting strategies, as well as quantify the degree of potential competition between cats, foxes and dingoes. Identifying potential environmental correlates associated with the amount of dietary overlap is informative in understanding how perturbances (such as decline of rabbit populations) are likely to influence food web dynamics in different ecosystems.
(5) Finally, we tested whether dietary composition is correlated with different sampling methods, to inform the protocols used to study and interpret predator diet.

## 2. Material and methods

### 2.1. Study species

#### 2.1.1. Domestic cat (3–7 kg)

The domestic cat was first introduced to Australia by Europeans in about 1788 (approx. 230 years ago) [42] and dispersed rapidly across the continent [43]. It is now present across the entire mainland and about 100 offshore islands [44]. Cats have been linked to the extinction of more than 20 native mammal species [14,45]. Evidence of cat impacts on extant fauna also comes from field experiments that recorded positive responses of small mammals to cat (and fox) population suppression [46–48] or complete exclusion [49,50]. Similarly, reintroductions of small- and medium-sized mammals have typically failed in open landscapes, but succeeded on islands and inside fence enclosures that are free of cats and foxes [51,52].

Cats are obligate carnivores [53], and typically prey upon small animals less than 200 g, although they can also capture prey up to their own body mass [54]. Cats are usually cited as only scavenging carrion as a response to a shortage of live prey [55,56], but consumption of livestock [57,58] and even dingo [59] carrion has been recorded, and they consume human refuse around disposal tips (e.g. [60]). In our study, we have included dietary data for feral and unowned stray cat populations (stray cat diet studies have been separately identified where they were conducted around refuse tips) but did not consider studies of pet cat diet.

Cats are solitary, mostly nocturnal hunters, employing a mobile strategy when visually seeking prey [61] or a stationary sit-and-wait (ambush) strategy at potentially productive areas, such as around prey refuges or feeding grounds [62]. Cat hunting strategies vary with prey type [62], with the mobile strategy likely to be most effective for small birds or reptiles, and the ambush strategy employed for fossorial prey, e.g. near rabbit warrens [55] and greater bilby (*Macrotis lagotis* (Reid)) burrows [63,64]. Although cats often hunt in open habitat, they can climb trees to target a suite of arboreal prey [65,66], and are small enough to enter rock crevices and burrows/warrens, to prey upon animals where they take refuge.

#### 2.1.2. Red fox (5–8 kg)

The fox was imported into southeastern Australia from Great Britain for sport hunting, becoming established from about 1874 [67] and subsequently spreading across about 80% of the continent [10,16] and onto *ca* 50 islands [16]. It is absent from northern arid regions and monsoonal tropics north of 18°S, and from Tasmania and Kangaroo Island [68,69]. The impacts of foxes on native fauna have been demonstrated from re-introduction programmes and removal experiments [70–72], which show that mammal recoveries are possible in the presence of intensive fox control, and by the recovery of many species following large-scale fox-baiting programmes [73–75].

Foxes are usually solitary hunters, employing an active searching method to locate food, regularly surveying their territories for food and making sorties into lesser-known areas in search of new food opportunities [76]. Mammals and invertebrates are the most common foods consumed in Australia, followed by birds and reptiles [23]. They also readily scavenge human refuse [77], fruit and crops [78] and carrion [79,80].

Like cats and dingoes, foxes make extensive use of rabbits as prey, and the spread of foxes across the continent followed the spread of rabbits with an approximate 10-year lag [10]. While some native animal declines preceded the arrival of foxes [81], range expansion of the fox coincided with local and regional declines of many medium-sized mammals, birds and freshwater chelid turtles [82]. Subsequently, foxes have been controlled across parts of their Australian range to mitigate threats to fauna as well as small livestock [69,83].

#### 2.1.3. Dingo (male weight 12–22 kg)

Since anthropogenic introduction, dingoes have established in most Australian mainland environments. They are absent from some larger islands including Tasmania, and Kangaroo, King and Flinders Islands, but are present on K'gari (Fraser Island), the Tiwi Islands and Groote Eylandt. Dingoes have been linked with faunal extinctions: their arrival on the Australian mainland preceding extinctions of the Tasmanian native-hen (*Gallinula mortierii* Du Bus) [84], the Tasmanian devil (*ca*. 3180 years BP) and the thylacine (*Thylacinus cynocephalus* (Harris), *ca*. 3230 years BP) [85]. However, there were also contemporaneous shifts in climate, human population density and human hunting techniques [86–88]. Dingoes have also been implicated in the declines of 10 medium-sized mammal species in central Australia since 1930 (e.g. common brush-tailed possum, *Trichosurus vulpecula* (Kerr) [89]), with predation pressure by dingoes increasing following the broadscale habitat modification from expansion of the pastoral industry and concurrent establishment of rabbits [90].

Dingoes are both solitary and social hunters, making them efficient and effective [91,92]. Dingoes can ambush prey (e.g. rabbits at warrens [93]), engage in active pursuit hunting strategies to chase down individual prey, or form groups to flush small animals or subdue large animals [90,91,94]. They also regularly consume human refuse [95,96] and carrion when available [97,98], especially kangaroos [99] and large animals such as camels *Camelus dromedarius* Linnaeus (e.g. [100]), horses *Equus caballus* Linnaeus [101] and at least some of the sheep and cattle (e.g. [91,102]) in their diets.

Dingoes can hybridize with modern dog breeds, and the genetic purity of dingoes varies across the continent [103,104]. For the purposes of our study, we have included dietary data for all free-roaming populations of dingoes, modern domestic dogs and their hybrids, hereafter collectively termed ‘dingoes', and have not tried to disentangle dietary studies according to the genetic purity of the source dingo population.

### 2.2. Literature search and data collection

**Figure 1.**
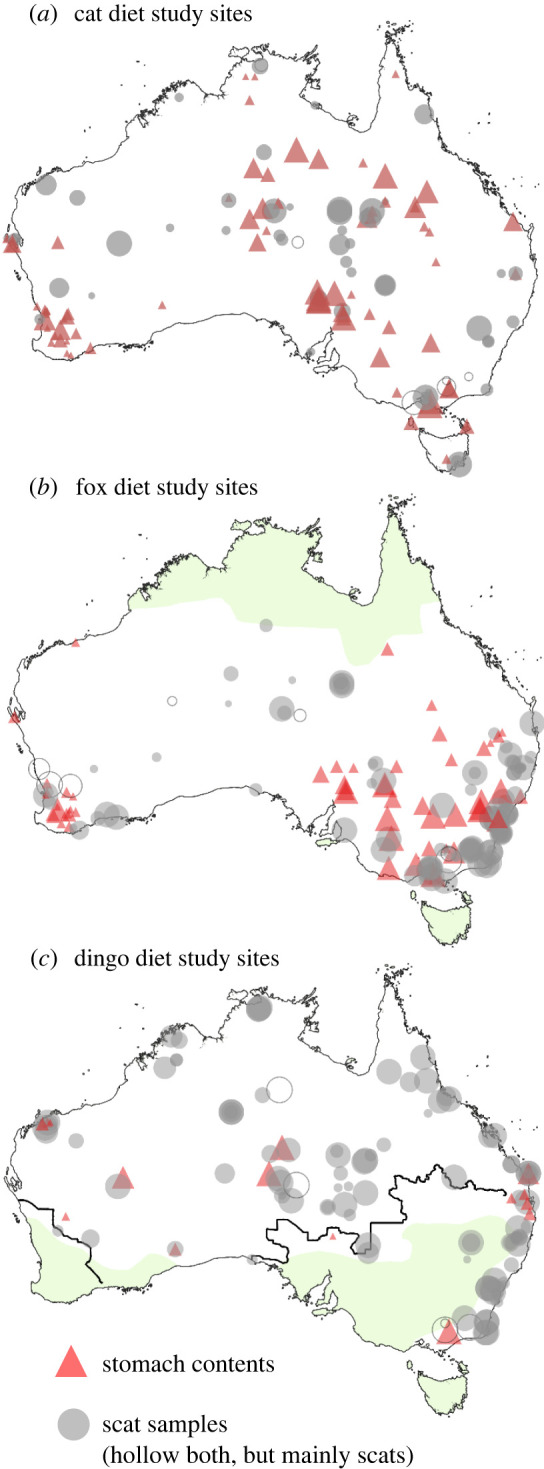
Locations of diet studies examined in this study for the domestic cat (*Felis catus*), red fox (*Vulpes vulpes*) and dingo (*Canis familiaris*) in Australia. Symbol size is proportional to the number of samples. Shaded areas represent absences/scarcity for foxes [16] and dingoes [83]. Studies of cat diet were somewhat uniformly distributed across the mainland and islands. Feral cats are found across the entire continent (except some predator free reserves). Foxes are largely absent from the northern tropics of the continent (green shaded area) and occur at greater densities in southwestern and southeastern forest areas as well as around cities [16]; as a natural consequence of their geographical range, there was a greater density of fox diet studies carried out in these general areas. The dingo has been removed from around sheep production landscapes (green shaded area) and accordingly there were few dingo diet studies in intensive agricultural zones southwest of the State Barrier Fence in Western Australia and south of the Dingo Barrier Fence (dark lines) where dingoes are either absent or scarce as a consequence of widespread intensive control/exclusion [83]. Both foxes and dingoes are absent from Tasmania.

We systematically searched the literature for empirical data of the FOO (the proportion of all samples that contain the diet item) of foods consumed by each of the three predator species (details in electronic supplementary material, appendix S1) and traced all citations for further potential sources, including journal articles, book chapters, theses, unpublished reports and contacted authors directly where possible for clarification and additional data. For the 157 studies using classical morphological methods (macro and microhistology) to identify diet items, we calculated FOO data for 421 location–time point combinations for 264 sites across Australia (figure 1) for:

(i) 7 main food categories: all mammals (summed), birds, squamate reptiles (squamates), amphibians (frogs), fish, invertebrates and plant material.
(ii) 15 broad mammal taxonomic groups (excluding instances of marine species in predator diets), including:
  — *Nine native mammal taxonomic groups*—dasyurids (Family Dasyuridae); possums and gliders (Suborder Phalangerida); macropods and potoroids (Suborder Macropodiformes); bandicoots and bilbies (Order Peramelemorphia); koalas *Phascolarctos cinereus* (Goldfuss); wombats (*Lasiorhinus* spp., bare-nosed wombat *Vombatus ursinus* (Shaw)); bats (Order Chiroptera); monotremes (short-beaked echidna *Tachyglossus aculeatus* (Shaw) and platypus *Ornithorhynchus anatinus* (Shaw)); and marsupial moles (*Notoryctes* spp.). Data were insufficient for the numbat (*Myrmecobius fasciatus* Waterhouse), which has a limited geographical range.
  — *Two introduced mammal categories*—lagomorphs (European rabbit and European brown hare, *Lepus europaeus* Pallas) and livestock. 'Livestock' included farmed (mostly sheep, cattle and goat *Capra hircus* Linnaeus) and feral livestock, some likely to have been scavenged (from most to least commonly recorded: sheep, cattle, feral pig *Sus scrofa* Linnaeus, camel, goat, sambar deer *Cervus unicolor* Kerr, chital deer *Axis axis* (Erxleben), horse, water buffalo *Bubalus bubalis* Linnaeus, fallow deer *Dama dama* (Linnaeus), hog deer *Axis porcinus* (Zimmermann), red deer *Cervus elaphus* Linnaeus and donkey *Equus asinus* Linnaeus).
  — *Rodents*—FOOs were mostly calculated separately for introduced and native species, but were analysed together because species were not always distinguished. Their separate FOO values contributed to the calculations of the FOO of native versus introduced species.
  — *The three predator species themselves*.

Where published studies included only data summaries (e.g. different size classes of mammals, or native versus introduced mammals), we sought primary data (i.e. records of mammal occurrence in individual scats or stomach samples) from the data custodians where possible. Where the raw data were unavailable, given that frequency values are not additive, we used the approach outlined in Murphy *et al*. [31] to sum FOO proportions for these broad taxonomic groups (see electronic supplementary material, appendix S1).

### 2.3. Data analyses

Data analyses were carried out in R (version 4.1.1; [105]). Data are presented as means±1 s.d. except where indicated.

#### 2.3.1. Do traits predict the likelihood of a species being recorded in a dietary study?

We analysed the traits of 225 mammal, 752 bird and 963 squamate species to assess the likelihood that they have been reported in the diets of cats, foxes or dingoes (from dietary studies, observations of predation events and species recovery plans) in 12 separate regression analyses. The dependent variables were the FOO (mammal prey species only) or the presence (presence/absence, where 1 = recorded in the diet versus 0 = not recorded in the predator's diet) of each prey species in predator diets. An important caveat of the presence/absence approach is that a species *recorded* in a diet can be influenced by multiple biases such as where diet studies have been conducted and how identifiable a prey species is from dietary samples. These values also do not directly reflect the numbers of animals killed, because some animals are killed but not eaten (i.e. surplus killing [10,106]), and some predators (especially foxes and dingoes) take substantial amounts of carrion that were not killed by these two predators themselves. As such, these model results should be interpreted as the likelihood of a species occurring in a diet study, rather than the likelihood of a species being killed or preyed upon by a predator species.

Predictor variables for each potential prey species (details in electronic supplementary material, appendix S2*a*) were (1) their adult body mass, (2) if they are active at night (yes, or no for diurnal active-only species), (3) if they are predominantly aquatic (yes or no), (4) relative cover of their favoured habitat (from 1 = gibber plain and rock to 6 = closed rainforest), (5) if they commonly forage on the ground (from 0 = never to 3 = feeds entirely on the ground), (6) if they take or seek refuge on the ground (yes, or no for arboreal, fossorial or saxicoline species). We also included surrogate measures of (7) relative abundance and distribution and (8) number of predator diet studies within each bioregion occupied by that prey species as covariates to account for the likelihood that each species might appear in predator diet studies.

FOO models were fitted using a Tweedie distribution, which applies a gamma distribution to the data that accounts for true zeros (absence of particular prey species), using the ‘tweedie’ package [107] in R. Presence/absence models were fitted using a binomial generalized linear model (*glm*) in the ‘lme4’ package [108] in R. The effects of predictor variables on diet overlap for the best model were visualized using *ggpredict* from the ‘ggeffects' package [109] in R, when all other variables are held constant at their mean/median value.

#### 2.3.2. Pianka's index of dietary overlap

For studies that presented simultaneously collected diet data on more than one predator species, we calculated dietary overlap, *O_ij_* [110], where 0 = entirely different foods are consumed and 1 = the diets are the same, from FOO data for each mammal species with other food categories pooled by broad grouping (as described *in Section 2.2* above) using the ‘spaa’ package [111] in RS2 (details in electronic supplementary material, appendix S3). We analysed overlap data using a generalized linear model in ‘lme4’ (followed by Tukey's post-hoc analysis using the ‘emmeans’ package [112]) for associations by predator species. Predictor variables (details in electronic supplementary material, appendix S2*b*) were (1) the pair of predators compared, (2) year of study, (3) whether the study was carried out during rodent irruption years, (4) mean of lagomorph FOO values across all predators reported within the study, (5) vegetation condition (from 1 = residual to 6 = removed), (6) percentage vegetation cover, (7) mean annual precipitation, and (8) mean annual temperature.

We tested for collinearity between predictors using the *vif* function in the ‘car’ package [113] in R. This test indicated that vegetation cover, which showed a strongly non-Gaussian distribution, was correlated with rainfall (*R_s_* = 0.888), temperature (*R_s_* = −0.746) and year of study (*R_s_* = −0.394); when vegetation cover was removed from the dataset all variance inflation factors were less than 1.6. Models including combinations of these predictors, either as additive or interacting factors, were compared using AIC to select the best model fit to the data, and predictors that were not retained in the top models (ΔAIC < 2) were excluded from further consideration. The effects of predictor variables on diet overlap for the best model were visualized using *ggpredict*, in which all other variables are held constant at their mean/median value.

#### 2.3.3. Diet composition and distinctiveness

We summed the FOO for each of the main food categories and each mammal species across all studies and ranked these from most to least commonly reported. We have separately reported those items that accounted for 80% of the cumulative FOO as a ‘top food list’ for cats, foxes or dingoes. We note that these values are indicative of how commonly each food type is reported, not the actual overall FOO in diet, as they do not take into account differences in sample size between studies.

To test for between-species differences in overall diet composition (including animals taken as either prey or carrion), we carried out non-parametric permutational ANOVA (PERMANOVA) using the *adonis* function in the ‘vegan’ package [114] in R. Owing to differences in reporting of diet and because this analysis requires a complete dataset, two separate analyses were carried out, comparing (i) 304 datapoints reporting the incidence of the 7 main food categories or (ii) 367 datapoints reporting the incidence of the 15 broad mammal taxa (as described in *Section 2.2* above). Predictor variables (details in electronic supplementary material, appendix S2*c*) were: (1) predator species, (2) sample type (0 = scats or 1 = stomach contents), (3) sampling location (mainland or island), (4) sampling location modification (natural or modified i.e. collected around a rubbish tip), (5) year of study, (6) vegetation condition, (7) percentage vegetation cover, (8) terrain ruggedness, (9) human population density, (10) distance to coast, (11) mean annual precipitation, and (12) mean annual temperature. To identify bias due to sample type, we carried out a Similarity Percentage (SIMPER) analysis on the differences between analyses of stomach contents and scats.

We then used the *multipatt* function in the ‘indicspecies’ package [115] in R to identify the diet categories most strongly associated with the diets of each predator or groups (pairs in this case) of predators.

#### 2.3.4. Mantel test to identify trends with spatial or temporal separation

A Mantel test was carried out to analyse spatial and temporal influences on diet composition for (i) the 7 main food categories and (ii) the 15 broad mammal taxa (as described in *Section 2.2* above). First, the analysis was carried out comparing the correlation between the difference in diet composition for pairs of studies against the Haversine distances between each pair calculated from their spatial coordinates. Second, the difference in diet composition for pairs of studies was compared against the time difference (year of study) between pairs of studies.

#### 2.3.5. GLM to compare FOO lagomorphs with time and RHDV

We carried out Spearman's rank order correlation analysis to identify which prey types changed in FOO over time. This was followed by a generalized linear model (GLM) with Tukey's post-hoc analysis (using the ‘emmeans’ package) to compare lagomorph FOO before and after the vectored introduction of the lagovirus, Rabbit Haemorrhagic Disease Virus (RHDV) in 1995 [116], and then after introduction of more lethal strains of the virus since 2015 [25,117]. The viral transmission routes between rabbits by oral, nasal and parenteral transmission via flies following dissemination by humans ensure rapid spread [117,118]; consequently, we made no attempt to time the spread of the virus in our comparison of predator diets. Studies that spanned dates for the introduction of lagovirus strains were not included in this GLM analysis.

## 3. Results

### 3.1. Which traits predict the likelihood of a species being recorded in the diet of cats, foxes or dingoes?

Of 1941 extant mammal, resident bird and squamate species in Australia, 196 mammal species (85% of described native species and 100% of introduced species), 396 bird species (52% of described native species and 73% of introduced species) and 274 squamate species (28% of described extant species; there are only eight introduced squamate species in Australia in our database) have been recorded in the diets of cats, foxes or dingoes. Of the total described species, 70% of mammals, 48% of birds and 27% of squamates have been recorded in the diet of cats, 53% of mammals, 18% of birds and 10% of squamates in the diet of foxes, and 66% of mammals, 7% of birds and 3% of squamates have been recorded in the diet of dingoes.

**Figure 2.**
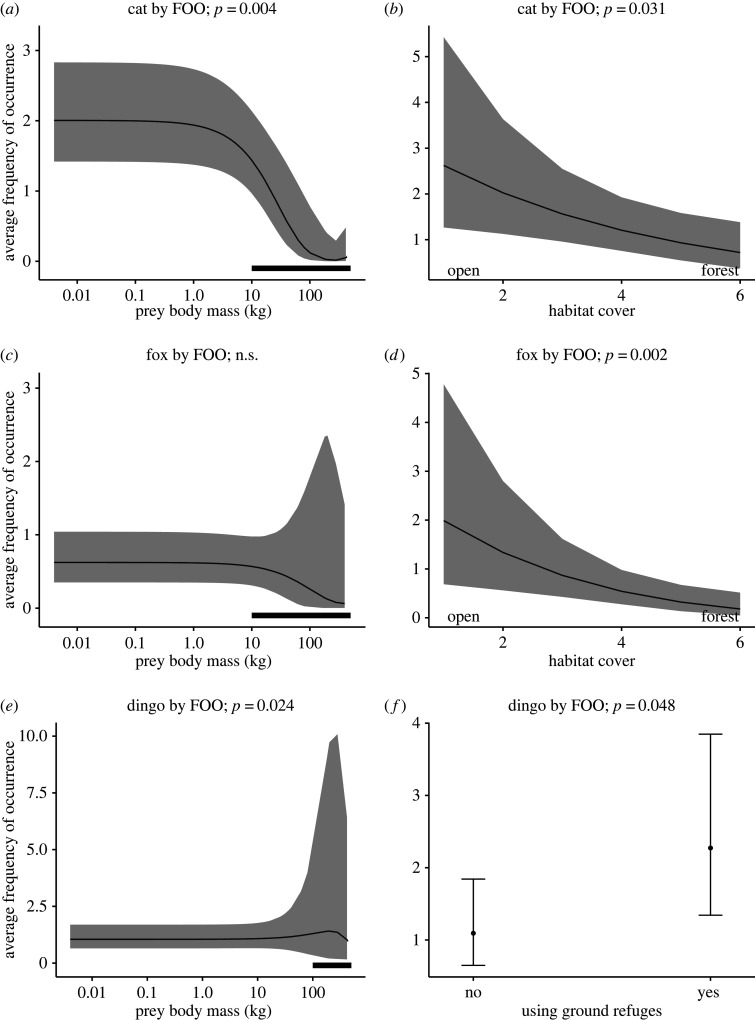
Mammal prey traits associated with their average FOO in diets of the (*a*,*b*) domestic cat (*Felis catus*), (*c*,*d*) red fox (*Vulpes vulpes*) and (*e*,*f*) dingo (*Canis familiaris*) in Australia. Predictions are from a regression analysis using the ggpredict function comparing the traits of individual mammal species with their average FOO in the diets of each predator, plotted with all other traits held at their mean or median values. Black lines against the *x*-axis for the left-hand panel indicate prey body masses that are likely to reflect scavenging of carrion by these predators.

Of the individual species’ traits, the strongest correlation with occurrence in predator diet studies for mammals, birds and squamates was their body mass (table 1 and figure 2; note the large variability for the largest mammal prey taken by both the fox figure 2*c* and dingo figure 2*e*, which were most likely taken as young animals or carrion—black bar along the *x-*axis). Ground-foraging and -nesting birds were more likely to appear in the diets of all three predators (*p* < 0.019, table 1).

**Table 1.** Summary of regression analysis comparing the traits of mammal, bird and squamate reptile species with their FOO (mammal species only) or likelihood that they are present in the diets of the introduced domestic cat (*Felis catus*), red fox (*Vulpes vulpes*) and dingo (*Canis familiaris*) in Australia. For each model, two predictors were included as surrogate measures of (1) relative abundance: Atlas of Living Australia (ALA) records, (2) range size: number of predator diet studies within the species' estimated range (results not shown). All predictors were mean-standardized before analysis, so that the estimates (Est.) are comparable between predictors. *p<0.05, **p<0.01, ***p<0.001.

	(a) cat				(b) fox				(c) dingo			
	Est.	s.e.	*z* value	*p*	Est.	s.e.	*z* value	*p*	Est.	s.e.	*z* value	*p*
mammals (average FOO)												
(intercept)	0.22	0.21	1.03	0.306	0.90	0.05	19.03	<0.001***	−0.16	0.25	−0.64	0.522
log-(body mass; kg)	−7.43	2.10	−3.54	<0.001***	−0.57	0.51	−1.13	0.262	5.00	2.20	2.27	0.024*
log-(body mass; kg)^2^	−6.14	2.13	−2.89	0.004**	−0.71	0.53	−1.33	0.184	−3.48	2.08	−1.68	0.095
habitat cover (0 = open to 6 = rainforest)	−0.37	0.17	−2.18	0.031*	−0.14	0.05	−3.12	0.002**	−0.14	0.20	−0.73	0.468
ground foraging (0 = no to 3 = always)	0.34	0.22	1.59	0.114	0.06	0.05	1.22	0.223	0.18	0.25	0.72	0.470
ground nesting/refuge (0 = no, 1 = yes)	0.42	0.25	1.70	0.090	0.06	0.07	0.80	0.422	0.63	0.31	1.99	0.048*
mammals (presence/absence)												
(intercept)	0.80	0.27	2.95	0.003**	−0.14	0.36	−0.39	0.695	0.91	0.28	3.30	0.001***
log-(body mass; kg)	−11.89	2.98	−3.99	<0.001***	−4.58	3.32	−1.38	0.168	−0.42	3.19	−0.13	0.894
log-(body mass; kg)^2^	−7.99	2.95	−2.71	0.007**	−4.49	3.21	−1.40	0.163	4.05	3.38	1.20	0.230
habitat cover (0 = open to 6 = rainforest)	−0.26	0.27	−0.98	0.329	−0.54	0.30	−1.79	0.074	−0.20	0.26	−0.76	0.449
ground foraging (0 = no to 3 = always)	−0.22	0.29	−0.77	0.443	0.00	0.42	0.00	0.998	0.24	0.29	0.83	0.405
ground nesting/refuge (0 = no, 1 = yes)	0.67	0.42	1.59	0.111	−0.77	0.50	−1.54	0.123	−0.63	0.41	−1.53	0.126
birds (presence/absence)												
(intercept)	−3.78	0.47	−8.12	<0.001***	−12.63	1.38	−9.16	<0.001***	−12.74	1.94	−6.56	<0.001***
log-(body mass; kg)	−3.81	2.46	−1.55	0.121	9.47	3.35	2.83	0.005**	15.15	4.40	3.44	0.001***
log-(body mass; kg)^2^	−3.71	2.38	−1.56	0.118	−2.42	3.47	−0.70	0.484	5.45	4.18	1.30	0.192
habitat cover (0 = open to 6 = rainforest)	−0.13	0.07	−1.98	0.048*	−0.06	0.10	−0.62	0.535	−0.01	0.15	−0.10	0.918
ground foraging (0 = no to 3 = always)	0.40	0.10	4.25	<0.001***	0.68	0.14	4.96	<0.001***	0.77	0.20	3.92	<0.001***
ground nesting/refuge (0 = no, 1 = yes)	0.73	0.19	3.84	<0.001***	1.63	0.27	6.12	<0.001***	0.85	0.36	2.34	0.019*
reptiles (presence/absence)												
(intercept)	−5.42	0.45	−12.11	<0.001***	−5.94	0.66	−9.04	<0.001***	−9.68	1.54	−6.29	<0.001***
log-(body mass; kg)	−1.21	2.96	−0.41	0.683	11.66	4.23	2.76	0.006**	23.88	8.04	2.97	0.003**
log-(body mass; kg)^2^	−0.92	3.06	−0.30	0.763	−3.84	4.17	−0.92	0.357	−3.91	6.39	−0.61	0.540
habitat cover (0 = open to 6 = rainforest)	0.01	0.07	0.14	0.888	−0.29	0.11	−2.70	0.007**	−0.51	0.20	−2.58	0.010**
active at night (0 = no, 1 = yes)	0.26	0.21	1.25	0.210	0.51	0.27	1.88	0.060	−1.34	0.54	−2.46	0.014*
ground nesting/refuge (0 = no, 1 = yes)	0.10	0.20	0.50	0.617	0.31	0.27	1.13	0.258	0.28	0.48	0.58	0.565

**Figure 3.**
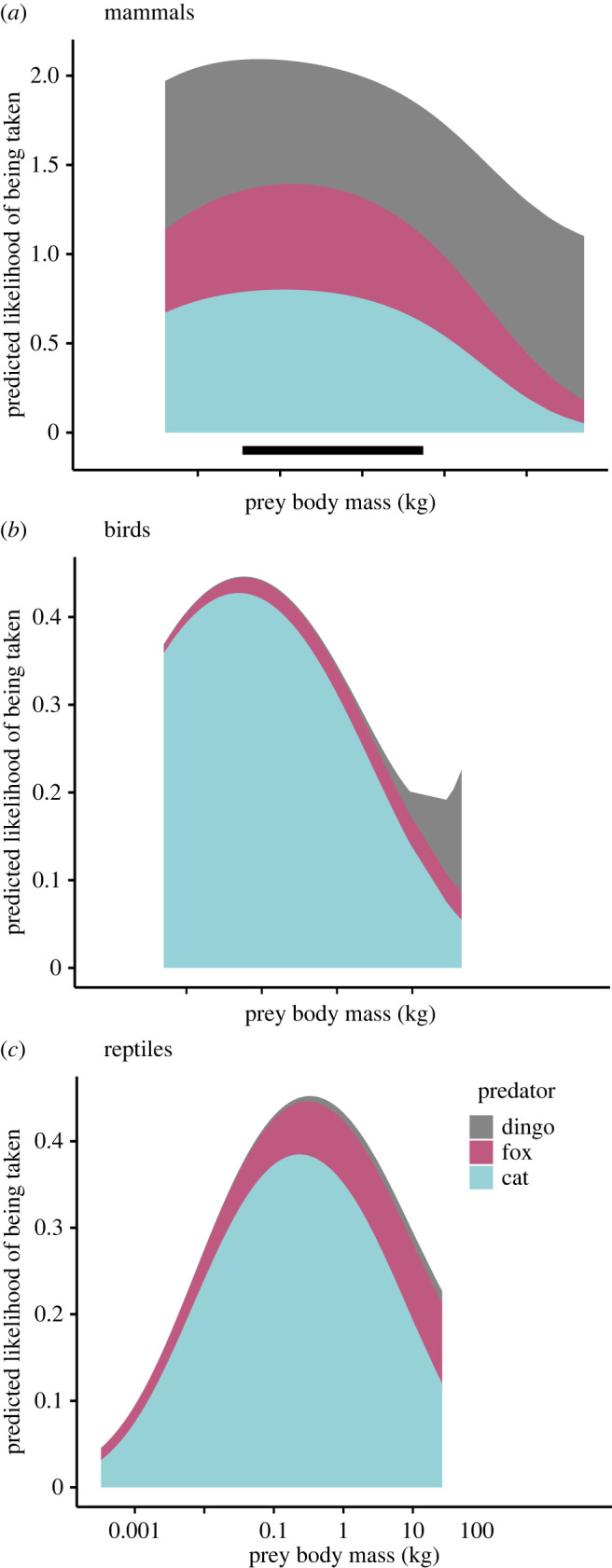
Cumulative predicted likelihood (based on presence/absence) for individual (*a*) mammal, (*b*) bird and (*c*) squamate reptile prey species being recorded in diet studies of the domestic cat (*Felis catus*), red fox (*Vulpes vulpes*) and dingo (*Canis familiaris*) in Australia, shown against the prey species' body mass. Predictions are from a logistic regression analysis using the *ggpredict* function comparing the traits of individual species with the likelihood that they have been reported present in the diets of one of the three predators, plotted with all other traits held at their mean or median values. Black line against the *x*-axis in (*a*) indicates the ‘critical weight range’ for mammals (35 g–5.5 kg that are now extinct or threatened [11–14]).

Quadratic and linear relationships were both significant predictors for the presence (*p* < 0.007, figure 3*a*) and FOO (*p* < 0.004, figure 2*a*) of mammal prey species in cat diets, with negative estimates for the linear relationships indicating the cat's preference for smaller prey. There were inverse relationships between habitat cover and the FOO of mammal prey (*p* = 0.031, figure 2*b*) and the presence of bird prey (*p* = 0.048), with cats consuming more mammal and bird species that used open habitats.

There was greater likelihood of larger bird (*p* = 0.005) and squamate (*p* = 0.006) prey in fox diet, but neither the presence (figure 3*a*) nor FOO (figure 3*c*) of mammal prey in fox diet showed a relationship with the prey species’ body mass. There were significantly greater FOO of mammal prey (*p* = 0.002, figure 2*d*) and greater likelihood of squamate species (*p* = 0.007) from more open habitats in fox diet.

While there was no significant relationship between the presence of mammal prey in dingo diet and the prey species' body mass, there was greater FOO of larger mammal prey (*p* = 0.024, figure 2*e*). There was also greater likelihood of larger bird (*p* = 0.001) and squamate (*p* = 0.003) prey in dingo diets. There was significantly greater FOO of mammal prey species using ground refuges in dingo diet (*p* = 0.048, figure 2*f*), and greater likelihood of diurnal squamates (*p* = 0.014) and squamates from more open habitat (*p* = 0.010).

### 3.2. Distinctive diets of cats, foxes and dingoes

Review of 157 diet studies resulted in data from 267 sites for the three predator species: cats (*n* = 69 diet studies spanning 55 years of collection, with 143 location/time points totalling 14 731 samples; 48% of studies analysed scats), foxes (*n* = 85 fox diet studies spanning 69 years of collection, with 166 location/time points totalling 35 176 samples; 65% of studies analysed scats) and dingoes (*n* = 59 diet studies spanning 53 years of collection, with 129 location/time points totalling 49 198 samples; 88% of studies analysed scats). These studies cover the geographical ranges of the three species in Australia (figure 1).

**Figure 4.**
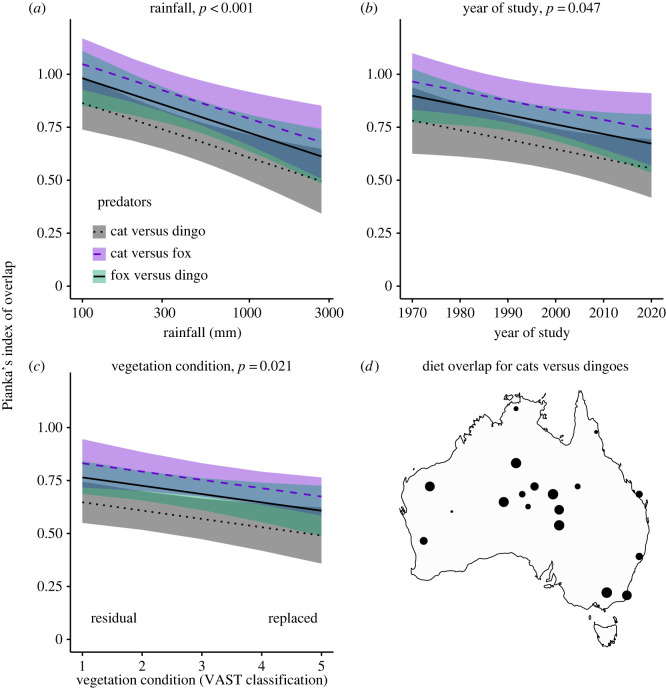
Summary of top factors correlated with diet overlap (Pianka's index of overlap) for pairs of predators, where 0 = entirely different foods consumed, and 1 = diet is the same. Lines show the predicted relationships (plus 95% confidence intervals) generated using *ggpredict*. Dots in (*d*) represent index of dietary overlap.

A subset of 46 studies presented dietary analyses that allowed calculation of dietary overlap between predators. The strongest model describing dietary overlap between pairs of predators is shown in table 2. The strongest factor influencing dietary overlap was the predator species being considered: the greatest dietary overlap was between cats and foxes (*O*_cf_ = 0.79 ± 0.20, *n* = 37 location/time points), an intermediate value for foxes and dingoes (*O*_fd_ = 0.73 ± 0.21, *n* = 42), and least overlap between cats and dingoes (*O*_cd_ = 0.64 ± 0.27, *n* = 24, figure 4*d*). Dietary overlap between predator species was greater in more arid sites (*p* = 0.001, figure 4*a*), for older studies (*p* = 0.047, figure 4*b*) and at sites with the most intact vegetation (*p* = 0.021, figure 4*c*). While lagomorph FOO was retained in the top models (*Δ*AIC < 2), it had weak relationship with dietary overlap values (table 2). Rodent irruption was not retained in the top models, with scarce data for times coinciding with rodent irruption.

**Table 2.** Summary of the best generalized linear model testing for relationships in diet overlap (Pianka's index of overlap) for pairs of predators. Vegetation cover and mean annual temperature were removed due to collinearity with other predictor variables; rodent irruption period (yes: greater than 50% FOO for one rodent species, or no: no rodent species had an FOO > 50%) and interaction terms were not retained in any of the top models (ΔAIC < 2). *p<0.05, **p<0.01, ***p<0.001.

	estimate	s.e.	*t*	*p*
(intercept)	0.61	0.04	13.91	<0.001***
predator pair (cat–dingo) - (cat–fox)	−0.18	0.06	−3.07	0.008**
(cat–dingo) - (fox–dingo)	−0.12	0.05	−2.14	0.087
(cat–fox) - (fox–dingo)	0.07	0.05	1.27	0.414
year of study	−0.05	0.03	−2.01	0.047*
lagomorph FOO	0.02	0.02	1.03	0.305
log (average annual rainfall)	−0.09	0.02	−3.46	0.001***
vegetation condition (VAST classification)	−0.06	0.02	−2.34	0.021*

**Figure 5.**
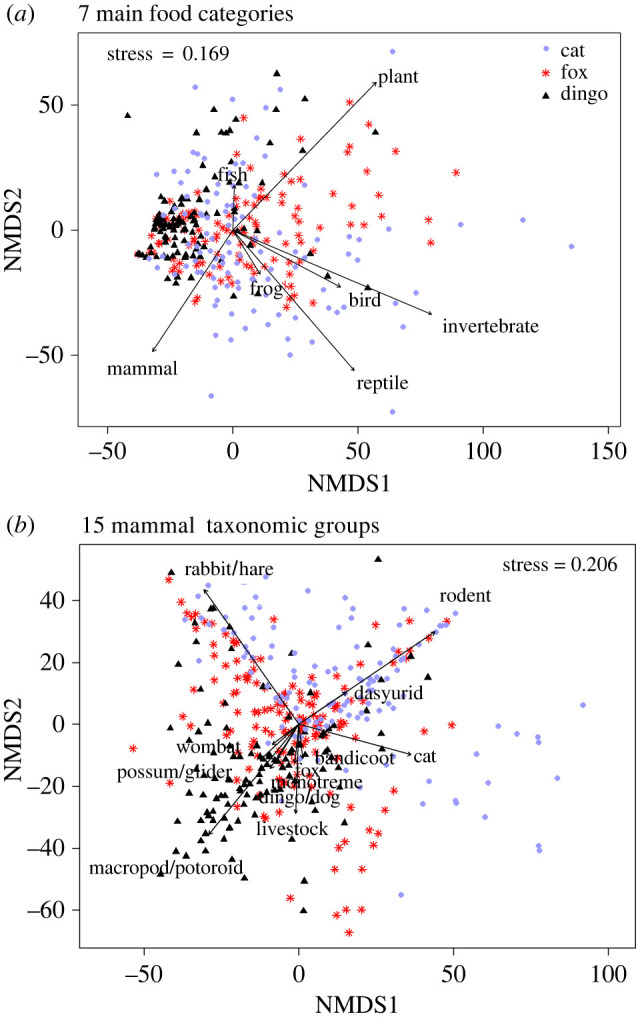
Nonmetric multidimensional scaling plots for dietary composition of the introduced domestic cat (*Felis catus*), red fox (*Vulpes vulpes*) and dingo (*Canis familiaris*) in Australia analysed as (a) seven main food categories and (b) 15 mammal taxonomic groups.

**Figure 6.**
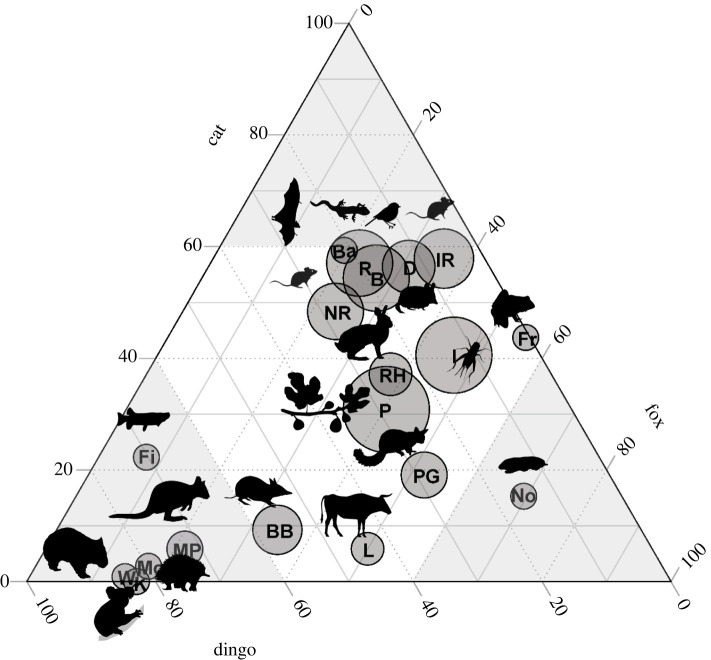
Ternary plot of diet composition for the domestic cat (*Felis catus*), red fox (*Vulpes vulpes*) and dingo (*Canis familiaris*) in Australia. Prey in the centre of the plot are consumed by all three species. Those towards each corner of the plot are consumed by one predator more than the other two. Relative size of circles represent relative total abundance in the diets of these three predators. Taxa shown are Ba, Bat; B, Bird; BB, Bandicoot; D, Dasyurid; Fi, Fish; Fr, Frog; I, Invertebrate; IR, Introduced Rodent; K, Koalas; L, Livestock; Mo, Monotreme; MP, Macropod/Potoroid; No, Marsupial moles; NR, Native Rodent; P, Plant; PG, Possum/Glider; R, Reptiles; RH, Rabbit/Hare; W, Wombat.

Similarly, although there were significant influences of environmental factors on diet composition, presumably reflecting ecogeographic factors determining prey distributions, the strongest factor associated with diet composition in nMDS analysis (figure 5) was predator species, when either the 7 main food categories (*p* < 0.001, table 3*a*) or 15 mammal taxonomic groups (*p* < 0.001, table 3*b*) were considered. FOO results for each diet category were used to construct a ternary plot showing patterns in diet composition for the three predators (figure 6), with food commonly consumed by all three species occupying the centre of the plot, and food categories more specific to each predator occupying the apices of the plot.

**Table 3.** PERMANOVA comparing diets of the introduced domestic cat (*Felis catus*), red fox (*Vulpes vulpes*) and dingo (*Canis familiaris*) in Australia for an analysis that included (*a*) the FOO of 7 main food categories, or (*b*) only 15 mammal categories. *p<0.05, **p<0.01, ***p<0.001.

	(*a*) 7 main food categories				(*b*) 15 mammal taxonomic groups			
	d.f.	F.Model	*R^2^*	*P*	d.f.	F.Model	*R^2^*	*p*
predator	2	38.79	0.13	<0.001***	2	46.10	0.13	<0.001***
sample type (propn. stomachs)	1	7.71	0.01	<0.001***	1	5.47	0.01	<0.001***
year of study	57	3.03	0.28	<0.001***	60	3.26	0.29	<0.001***
mainland versus island	1	10.09	0.02	<0.001***	1	3.17	<0.01	0.010**
modified (refuse tip versus natural)	1	9.58	0.02	<0.001***	1	1.82	<0.01	0.091
VAST classification	1	1.41	<0.01	0.240	1	2.80	<0.01	0.016*
percentage vegetation cover (5 km)	1	0.68	<0.01	0.611	1	12.13	0.02	<0.001***
terrain ruggedness (5 km)	1	4.35	0.01	0.007**	1	2.19	<0.01	0.046*
human population density (5 km)	1	4.58	0.01	0.006**	1	6.27	0.01	<0.001***
distance to coast (km)	1	5.14	0.01	0.002**	1	12.61	0.02	<0.001***
mean annual precipitation (mm)	1	12.92	0.02	<0.001***	1	13.14	0.02	<0.001***
mean annual temperature (°C)	1	14.46	0.02	<0.001***	1	16.64	0.02	<0.001***
predator × year of study	56	1.42	0.13	0.002**	60	1.12	0.10	0.126
residuals	211	0.34			254	0.37		
total	336				386

#### 3.2.1. What makes cats' diet distinctive?

Nine food items cumulatively made up 80% of the cat's diet (electronic supplementary material, appendix S4). From most to least commonly consumed prey groups, these were invertebrates (30 ± 20% FOO averaged across *n* = 97 studies/timepoints reporting their presence in the 113 total studies/timepoints analysed in this way for cat diet), birds (27 ± 20%, *n* = 97), squamates (25 ± 20%, *n* = 87), rabbit^†^ (29 ± 24%, *n* = 72), plant material (23 ± 17%, *n* = 71), house mouse *Mus musculus* Linnaeus^†^ (20 ± 18%, *n* = 76), long-haired rat *Rattus villosissimus* Waite (35 ± 28%, *n* = 16), common ringtail possum *Pseudocheirus peregrinus* Boddaert (26 ± 27%, *n* = 15), and black rat *Rattus rattus* Linnaeus^†^ (13 ± 12%, *n* = 28). ^†^ *indicates introduced species*. See electronic supplementary material, appendix S4 for averages across all studies/timepoints.

Of the 7 main food categories (table 4*a*), three were associated with the distinctiveness of cat diets. Birds (*p* < 0.001 for Indicspecies ‘indicator value’ statistics as shown in table 4) and squamates (*p* < 0.001) were found in greater numbers in cat diets. Cats were twice as likely to have consumed birds than foxes and 2.9 times more likely than dingoes. Cats were 2.5 and 2.9 times more likely to have consumed squamates than foxes and dingoes, respectively. These dietary items appear towards the apex of the ternary plot (figure 6), which captures the relatively greater incidence of these foods in cat diets. Furthermore, cat diets were distinguished from those of foxes and dingoes by lower mammal FOO (all mammals summed together; *p* < 0.001).

**Table 4.** FOO of each diet category where shaded cells represent categories that were distinctive to the diets of the introduced domestic cat (*Felis catus*), red fox (*Vulpes vulpes*) and dingo (*Canis familiaris*) in Australia according to the indicspecies statistics for (*a*) 7 main food categories and (*b*) 15 mammal taxonomic groups. Indicspecies analysis required no missing data in the dataset, and therefore rodents were analysed as both native and introduced species pooled together. Right-hand columns represent the change in FOO of that prey type over time (midpoint year of study). Cells are shaded blue = positive correlations (i.e. more of that diet category for more recent studies), to red = negative correlations (i.e. less of that diet category for more recent studies). *p<0.05, **p<0.01, ***p<0.001; ^†^indicates introduced species.

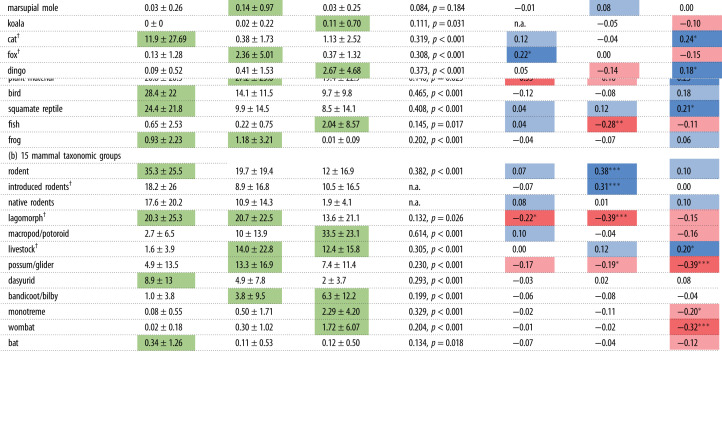

Of the 15 mammal taxonomic groups (table 4*b*), six were associated with the distinctiveness of cat diets. Cats were 1.8 and 2.9 times more likely to have consumed rodents (introduced and native analysed together) (*p* < 0.001), 1.8 and 4.5 times more likely to have consumed dasyurids (*p* < 0.001), and 3.0 and 2.8 times more likely to have consumed bats than foxes or dingoes (*p* = 0.018). By contrast, livestock were 8.5 and 7.5 times more frequent in fox and dingo diets (*p* < 0.001) and bandicoots/bilbies were 3.7 and 6.1 times more frequent in fox and dingo diets (*p* < 0.001). Cats had the greatest frequency of cat in their diets (*p* < 0.001), presumably representing the presence of grooming hairs.

#### 3.2.2. What makes foxes' diet distinctive?

Eleven food items cumulatively made up 80% of the fox's diet (electronic supplementary material, appendix S4). From most to least commonly consumed prey groups, these were invertebrates (38 ± 23% FOO averaged across *n* = 104 studies/timepoints reporting their presence in the 135 total studies/timepoints analysed in this way for fox diet), plant material (29 ± 23%, *n* = 92), rabbit^†^ (21 ± 21%, *n* = 125), sheep^†^ (24 ± 28%, *n* = 66), birds (13 ± 10%, *n* = 107), common ringtail possum (15 ± 16%, *n* = 72), house mouse^†^ (11 ± 12%, *n* = 91), squamates (12 ± 16%, *n* = 79), swamp wallaby *Wallabia bicolor* (Desmarest) (11 ± 11%, *n* = 61), common brushtail possum (9 ± 11%, *n* = 72), and bush rat *Rattus fuscipes* (Waterhouse) (12 ± 14%, *n* = 53). ^†^ indicates introduced species. See electronic supplementary material, appendix S4 for averages across all studies/timepoints.

Of the 7main food categories, only one was distinctive to fox diets: the incidence of plant material was 1.3 and 1.4 times greater in fox diet than cat or dingo diets (*p* = 0.025). Of the 15 mammal taxonomic groups, only three were associated with the distinctiveness of fox diets. Foxes were 2.7 and 1.8 times more likely to have consumed possums/gliders than cats and dingoes (*p* < 0.001). Although foxes were 4.5 and 4.6 times more likely to have consumed marsupial moles than cats and dingoes, the difference was not significant (*p* = 0.184), probably because these were reported in few studies. These prey taxa are plotted towards the bottom right corner of the ternary plot (figure 6), signifying the relatively greater incidence of these foods in fox diets. Foxes had a greatest frequency of fox in their diets (*p* < 0.001), presumably representing the presence of grooming hairs or possible cannibalism.

#### 3.2.3. What makes dingoes' diet distinctive?

Eighteen food items made up 80% of the dingo's diet (electronic supplementary material, appendix S4). From most to least commonly consumed prey groups, these were plant material (24 ± 24% FOO averaged across *n* = 83 studies/timepoints reporting their presence in the 132 total studies/timepoints analysed in this way for dingo diet), rabbit^†^ (19 ± 22%, *n* = 101), swamp wallaby (24 ± 17%, *n* = 68), invertebrates (13 ± 14%, *n* = 90), birds (11 ± 10%, *n* = 98), red kangaroo *Osphranter rufus* (Desmarest) (24 ± 25%, *n* = 41), squamates (12 ± 16%, *n* = 81), cattle^†^ (11 ± 16%, *n* = 83), long-haired rat (22 ± 26%, *n* = 24), common brushtail possum (8 ± 11%, *n* = 59), house mouse^†^ (7 ± 12%, *n* = 58), eastern grey kangaroo *Macropus giganteus* Shaw (7 ± 9%, *n* = 54), bare-nosed wombat (12 ± 10%, *n* = 30), agile wallaby *Notamacropus agilis* (Gould) (31 ± 20%, *n* = 11), northern brown bandicoot *Isoodon macrourus* (Gould) (12 ± 14%, *n* = 26), common ringtail possum (8 ± 8%, *n* = 41), euro *Osphranter robustus* (Gould) (8 ± 10%, *n* = 35) and echidna (4 ± 4%, *n* = 73). ^†^indicates introduced species*.* See electronic supplementary material, appendix S4 for averages across all studies/timepoints.

Of the 7 main food categories, three were associated with the distinctiveness of dingo diets. Dingoes were 3.2 and 9.5 times more likely to have consumed fish than cats and foxes (*p* = 0.017). By contrast, invertebrates were 3.0 and 3.4 times more frequent in cat and fox diets ( p < 0.001) and frogs were 64.7 and 82.2 times more frequent in cat and fox diets ( p < 0.001).

Of the 15 mammal taxonomic groups, six were associated with the distinctiveness of dingo diets. Dingoes were 12.5 and 3.4 -times more likely to have consumed macropods/potoroids than cats and foxes (*p* < 0.001), 29.0 and 4.5 times more likely to have consumed monotremes (*p* < 0.001), and 95.6 and 5.7 times more likely to have consumed wombats (*p* < 0.001). While we found no record of cats consuming koalas, dingoes were 4.9 times more likely to have consumed koalas than foxes (*p* < 0.031). These prey taxa are plotted towards the bottom left corner of the ternary plot (figure 6), signifying the relatively greater incidence of these foods in dingo diets. Cats and foxeswere 1.5 times more likely to have consumed lagomorphs than dingoes (*p* = 0.026). Dingoes had the greatest frequency of dingo in their diets (*p* < 0.001), representing both the presence of grooming hairs and cannibalism (e.g. [96,119]).

The average occurrence of native mammals (FOO *c*: 32 ± 31%, *f*: 34 ± 28%, *d*: 54 ± 22%) was 1.7 and 1.6 times greater in dingo diets than the average for cats and foxes, respectively, while the occurrence of introduced mammals (FOO *c*: 36 ± 28%, *f*: 39 ± 28%, *d*: 26 ± 24%) was 1.3 times greater in cat and fox diets than for dingo diets. Introduced and native rodents were reasonably equally represented in diets of cats and foxes, but dingoes consumed relatively more native than introduced rodents (table 4).

### 3.3. Spatial differences in diet composition

Mantel test results indicated significant correlations with spatial separation (the Haversine distance) for cat and fox diets for both the 7 main food categories and the 15 mammal taxonomic groups (table 5). Thus, the dietary composition of cats and foxes from adjacent study sites was more similar than samples collected from sites that were far apart, suggesting that their diets were influenced by geographical patterns in prey availability. Spatial differences were strongest for the mammal composition of fox diets. The association was only significant for mammal prey in dingo diet.

**Table 5.** Summary of spatial and temporal separation effects on diet composition (values presented are Mantel statistic correlation coefficients) analysed as (*a*) 7 main food categories and (*b*) 15 mammal taxonomic groups diet composition for the introduced domestic cat (*Felis catus*), red fox (*Vulpes vulpes*) and dingo (*Canis familiaris*) in Australia. *p<0.05, **p<0.01, ***p<0.001.

	cat	fox	dingo
(*a*) 7 main food categories			
spatial pattern	*r* = 0.068, *p* = 0.037*	*r* = 0.074, *p* = 0.033*	*r* = 0.038, *p* = 0.177
temporal pattern	*r* = 0.060, *p* = 0.047*	*r* = 0.130, *p* < 0.001***	*r* = –0.033, *p* = 0.793
(*b*) 15 mammal taxonomic groups			
spatial pattern	*r* = 0.227, *p* < 0.001***	*r* = 0.310, *p* < 0.001***	*r* = 0.105, *p* = 0.004**
temporal pattern	*r* = 0.046, *p* = 0.074	*r* = 0.118, *p* < 0.001***	*r* = 0.002, *p* = 0.469

### 3.4. Temporal patterns in diet composition

There was a significant effect of the *year of study* for both the 7 main food categories PERMANOVA (*p* < 0.001; table 3*a*) and the 15 mammal taxonomic groups (*p* < 0.001; table 3*b*), and a significant *predator x year of study* interaction for the 7main food categories (*p* = 0.002; table 3*a*) indicating different responses in dietary composition over time between the three predators.

There was some change in cat diet over time for the 7 main food categories (Mantel tests: table 5*a*), with notable decrease in the amount of plant material recorded in cat diets (pairwise correlations: table 4*a*). Although there was a decrease in the amount of lagomorph and an increase in the amount of fox recorded in cat diets over time (table 4*b*), the overall mammal prey composition of cat diets did not change significantly over time (table 5*b*).

There were significant changes over time in fox diet composition (Mantel tests, table 5), with the greatest dietary dissimilarity for studies that were more separated in time. More recent studies of fox diet found a lower incidence of lagomorphs and possums/gliders, but more mammals (generally) and rodents (particularly introduced rodents; table 4). There was also less fish recorded, although this category was scarce in fox diets.

Although there was no effect of temporal separation on overall dingo diet composition (Mantel tests, table 5), there were significant changes for some individual food items. There were fewer possums/gliders, monotremes and wombats recorded in dingo diet over time, but more livestock, invertebrates, squamates, plant material, cat and dingo.

**Figure 7.**
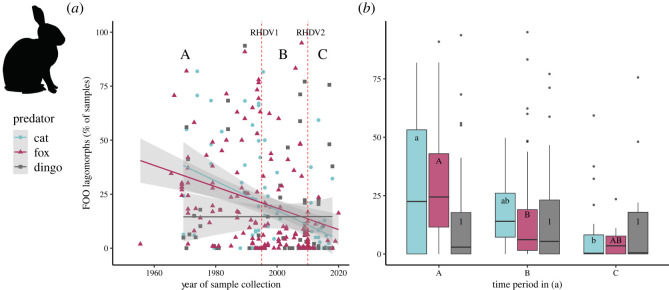
(*a*) Change over time in the FOO of lagomorphs (principally European rabbit *Oryctolagus cuniculus*) in the diets of the domestic cat (*Felis catus*), red fox (*Vulpes vulpes*) and dingo (*Canis familiaris*) in Australia. (*b*) Data shown by periods before and then after the introduction of Rabbit Haemorrhagic Disease Virus (RHDV) in 1996 [65,120], and then after introduction of more lethal strains of the virus since 2010 [121,122] (vertical lines in (*a*)). Studies that spanned the introduction of RHDV were not included in (*b*). Letters in (*b*) link time periods that are not significantly different (Tukey's post-hoc analysis following a generalized linear model).

Notably, lagomorph FOO decreased over time in the diets of both cats (*R_s_* = −0.220, *p* = 0.017) and foxes (*R_s_* = −0.390, *p* < 0.001), but there was no significant change over time for dingoes (*R_s_* = −0.150, *p* = 0.115) (figure 7*a*; table 4). Lagomorph FOO was less post RHDV2 than pre-RHDV (*p* = 0.007) in cat diet, and less post RHDV1 than pre-RHDV for fox diet (*p* = 0.009) (figure 7*b*), while no change was evident for dingo diets across these three time periods (figure 7*b*).

### 3.5. Biases due to sample type and sampling location

The PERMANOVA of dietary composition indicated an effect of *sample type* (i.e. stomachs or scats analysis, *p* < 0.001 for both the 7 main food categories and the 15 mammal taxonomic groups; table 3), likely reflecting differences in digestibility of food items. For the 7 main food categories, SIMPER analysis indicated significantly more plant (*p* = 0.001) and frog (*p* = 0.001) recorded from stomach contents, but more mammal recorded from scat material (*p* = 0.005), and analysis of the 15 mammal taxonomic groups indicated significantly more livestock (*p* = 0.001) and cat (*p* = 0.001) recorded from stomach contents (electronic supplementary material, appendix S5).

There were differences in dietary composition for mainland versus island (*p* < 0.001 for the 7 main food categories, table 3*a*; *p* = 0.010 for the 15 mammal taxonomic groups, table 3*b*) with predator diets from islands containing less mammal (average FOO mainland: 71 ± 19%, island: 53 ± 23%) and more bird prey (mainland: 16 ± 15%, island: 40 ± 30%) as well as a shift away from native mammals (mainland: 41 ± 29%, island: 17 ± 24%) to relatively more introduced mammals (mainland: 34 ± 27%, island: 36 ± 30%).

Finally, there was also an effect of modified sample sites (i.e. natural habitat versus human refuse tips) for the 7 main food categories (*p* < 0.001; table 3*a*), with fewer mammals (natural: 71 ± 19%, modified: 50 ± 27%) but more plant material (natural: 22 ± 22%, modified: 39 ± 24%) recorded in the diets of predators captured around human refuse tips.

## 4. Discussion

Our analysis of approximately 100 000 stomach and scat samples collated from 267 sites in 157 studies reveals predation by the three eutherian predators on 866 Australian native and introduced mammal, bird and squamate species (from a total of 1941 described species). Most Australian mammal species (86% of described species), over half of bird species, and more than a quarter of all squamate species have been recorded in the diets of the cat, fox or dingo. The strongest factor correlated with occurrence in predator diet studies (when all other traits were considered simultaneously) was prey body mass. While there was substantial overlap in cat, fox and dingo diets, with many species consumed by all three predators, there were differences in composition of their diets that likely reflect the predators' relative body sizes and hunting strategies, e.g. ambush versus active foraging, whether the predator is more likely to climb or dig out prey, and their readiness to consume carrion. Understanding these differences is informative in predicting the likely conservation impacts of these eutherian predators on prey.

### 4.1. Prey taken by cats

In Australia, cats are estimated to kill and eat 272 million birds each year [27], including 24 species listed as threatened or extinct by the IUCN [29], and our analyses identified that the large amount of bird prey was a distinctive characteristic of cat diets. While a few mainland studies recorded high incidence of birds in cat diets (e.g. FOO 83.1% [123]), the predatory impact of cats has been particularly marked on island bird populations (e.g. FOO > 70% [56,124–126]). The vulnerability of ground-foraging and -nesting bird species makes them of particular conservation concern [127,128].

Cat diets were also distinctive for the high incidence of squamate prey they had consumed. Several studies report squamates in more than two-thirds of cat diet samples for arid environments and islands [124,125,129,130]. Extrapolating from diet studies, it has been estimated that feral cats in Australia kill 466 million reptiles every year, with the greatest impact on intermediate-sized arid-zone species [28]. We found squamate prey occupying more open habitats more likely to be recorded in cat diets, which is likely to reflect cats' increased hunting efficiency in simplified vegetation structure [131–133].

In this study, only nine food items made up 80% of the cat's diet. However, four of the first five categories were pooled taxa: invertebrates, birds, squamates and plant material. To allow consistent comparison between predator diets, we could not separate these groups out to prey species. Although cats do eat plant material to aid digestion of heavy/coarse foods, they cannot taste sugar and have a limited ability to extract nutrition from cellulose. The high occurrence of plant material in cat diets is likely to be a legacy of accidental consumption with prey, eating plants as a digestive aid, or from eating the gut contents or whole body of small herbivorous/omnivorous species, rather than consumption of plants specifically for nutrition e.g. fruit and crops by foxes and dingoes.

The mammals most commonly consumed by cats were three widespread invasive species (rabbit, house mouse and black rat) and two native species that can be locally common (long-haired rat and common ringtail possum). The distribution of cat-prey body sizes indicated that many small mammal species are vulnerable to cat predation [35], and while mammals occurred less frequently in cat diets overall, cat diet included more small mammals than foxes and dingoes, particularly rodents (e.g. FOO *Notomys alexis* Thomas 13 ± 16% *n* = 17 studies/timepoints reporting their presence, *Pseudomys hermannsburgensis* (Waite) 11 ± 15% *n* = 21, *P. desertor* Troughton 10 ± 12% *n* = 11, bush rat 16 ± 11% *n* = 11, and *Leggadina forresti* (Thomas) 8 ± 10% *n* = 21) and small dasyurids (e.g. *Sminthopsis macroura* (Gould) 9 ± 16% n = 19, *S. crassicaudata* (Gould) 8 ± 10% n = 12*, Antechinus stuartii* Macleay 16 ± 9% n = 6*, Planigale ingrami* Thomas 13 ± 14% n = 7).

**Figure 8.**
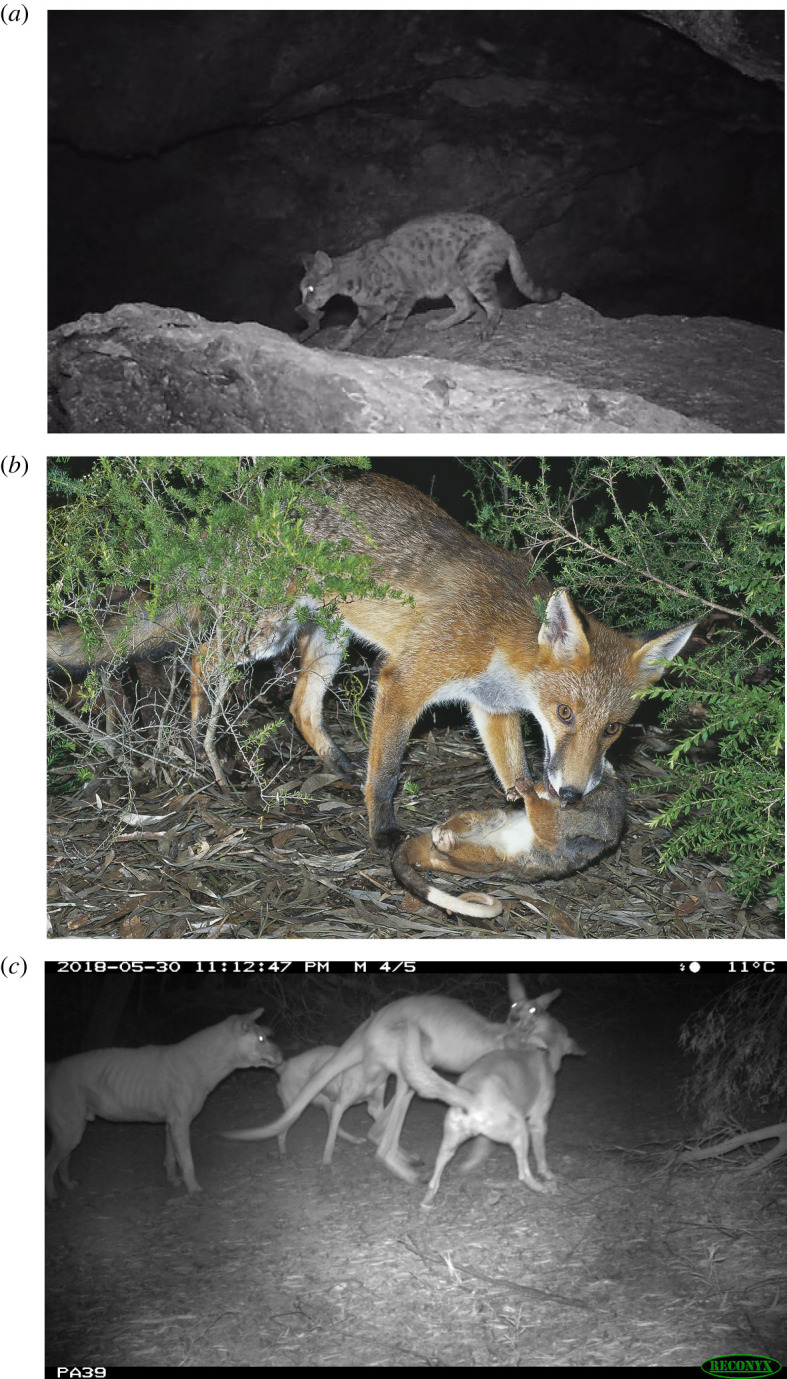
Prey types that are distinctive to the diets of the domestic cat (*Felis catus*), red fox (*Vulpes vulpes*) and dingo (*Canis familiaris*) in Australia. Images show (*a*) predation on Pilbara leaf-nosed bat (*Rhinonicteris aurantia (Pilbara form) (J.E. Gray)* ) by a feral cat (Photo credit Biologic Environmental Survey), (*b*) common ringtail possum (*Pseudocheirus peregrinus*) by fox (Photo credit Pavel German, australiannature.com) and (*c*) red kangaroo (*Osphranter rufus*) by a group of dingoes (Photo credit Peter Adams, Western Australia Department of Primary Industries and Regional Development).

Although cats occur and hunt in all environments or landforms [44,64], we found greater FOO of mammal species occupying more open habitats. Their ambush hunting behaviour is particularly well suited to catching small animals as they emerge from refuges [62,134] or vegetation cover [64]. Cats also hunt at animal refuges, for example, spending much time around small mammal burrows, and most time around rabbit and bettong warrens [64]. Although bats are relatively uncommon prey overall, their occurrence was higher in cat diet than in the diets of foxes and dingoes. The exploitation of bats by cats may encompass opportunistic hunting when bats are flying close to the ground (P.J.S.F., personal observation, 1979, Kingstown NSW) or, more usually, hunting at breeding bat roosts in buildings, trees or caves [135,136] (figure 8*a*).

### 4.2. Prey taken by foxes

Foxes have a diverse, generalist diet [137–139], opportunistically taking both live prey and carrion of different sizes and types [23,79,140], and feeding on substantial amounts of plant material and invertebrates. Of the 11 food items cumulatively making up 80% of the fox's diet, invertebrates were the most frequently recorded category, while the high incidence of plant material was distinctive to fox diet. Foxes consume substantial amounts of fruit and play a role in dispersing invasive weeds (e.g. [141,142]), although fruit is often not itemized separately or described in detail in predator dietary studies. Both invertebrates and fruit provide foxes with water, fat and sugar, which improve body condition and survival during lean times [143,144].

Foxes were likely to have consumed mammals across the entire range of body mass, from small, presumably live, prey up to the largest prey likely taken as carrion. Rabbits were the most frequently consumed mammal prey of foxes, with several studies reporting rabbits in greater than 80% of samples [90,145,146]. The incidence of rabbit in fox diet has significantly decreased over time [23] as successful biocontrol of these invasive animals has been implemented [147]. In the short term, foxes have been shown to switch to invertebrates and carrion after removal of rabbits [65,148] while, over the scale of decades, there has been increased frequency of introduced rodents in their diet.

The diet of foxes showed the greatest spatial and temporal variation of the three predators examined. The high incidence of possums and gliders was distinctive to fox diet; for example, common ringtail possum (figure 8*b*) and common brushtail possum were both on the top food list. Several studies report these marsupials in over half of all fox diet samples [58,149–151]. The consumption of possums was most marked across southeastern Australia [23] where the density of foxes is highest [16] and where possums are most abundant. For example, an average of 23% of all fox diet samples from sites across Victoria contains evidence of possums or gliders. However, we found a decline in the incidence of possums/gliders in fox diet over time. These mostly arboreal marsupials are extremely vulnerable when moving on the ground between trees, reflecting an indirect impact of the loss of continuous canopy due to clearing of vegetation or in naturally low or sparse vegetation where there is insufficient elevated canopy or avenues for escape [152]. The reduction in possum/glider prey over time could also reflect possum decline due to loss of large old trees [153] that provide important breeding habitats for these animals.

While we found no indication that body mass influenced the FOO or the presence of mammal species as prey in the diet of foxes, foxes were more likely to consume large squamates (e.g. *Varanus* spp.) and bird species. For example, foxes have had substantial impact on ground-nesting endangered bird species (e.g. hooded plovers, *Thinornis rubricolli* (Vieillot) [154]) and common but locally valued populations of other species (e.g. little penguins, *Eudyptula minor* (J.R. Forster), and short-tailed shearwaters, *Puffinus tenuirostris* Temminck [155] on Phillip Island, Victoria). The impacts of foxes on populations of susceptible ground-foraging and -nesting prey is likely to compound threats by co-occurring cats, dingoes and anthropogenic activities.

The fox's generalist diet means that their niche space is inherently plastic, changing in response to available food sources [156]. Consequently, foxes show strong dietary overlap with both cats and dingoes, with fewer dietary categories identified as distinctive to fox diets. The strong overlap in fox diet with the diets of both cats and dingoes shows that foxes target similar prey as cats, such as house mouse (included in both the fox and cat top food lists, e.g. [57,124]) and dasyurids (e.g. [157,158]), but are also large enough to target some prey preferred by dingoes, such as swamp wallabies (included in both the fox and dingo top food lists, e.g. [149,159]) and rock wallabies, *Petrogale* spp. [71,160].

### 4.3. Prey taken by dingoes

Although plant, invertebrate, bird and squamate categories were included in the top food list making up 80% of the dingo diet, their consumption did not comprise the majority of the dingo's diet with three introduced and 11 native mammal species also included on the top food list. This finding supports the description of dingoes as generalist predators (e.g. [19,90,94,161]), although the greater incidence of larger mammal, bird and squamate prey in their diet suggests they are selective where their preferred prey is present.

We found no evidence of long-term temporal variation in dingo diet composition, likely reflecting that their principally macropod diet has been fairly constant over time. Although rabbit was the single most commonly consumed mammal, the average FOO of lagomorphs in dingo diet was lower than for cat and fox diets, and there was no significant change in frequency of rabbits in dingo diet over time. There was no evidence of spatial differentiation in dingo diet for the 7 main food categories, although there was a pattern for their mammal prey, reflecting that dingoes show dietary opportunism in response to localized prey availability.

Analysis of the traits of mammal prey that appear in dingo diets uncovered no relationship with prey body mass, with small species such as native rodents (e.g. [100,162]) appearing in dingo diets as well as larger mammals, such as kangaroos. However, this traits analysis does not reflect the numbers of prey consumed, as commonly consumed species each only contribute a single datapoint in this analysis. By contrast, analysis of FOO of prey indicates that dingoes are far more likely to take larger prey (e.g. [140]), including farmed and feral livestock [101] as well as kangaroo, wallaby and potoroid species (Macropodiformes). The dominance of macropods in dingo diets has been widely reported (e.g. [91,102,158,163,164]). While most macropods eaten by dingoes are common species (contributing to the dingo top food list: swamp wallaby, red kangaroo (figure 8*c*), eastern grey kangaroo, agile wallaby, euro), dingoes also consume some locally rare or threatened native species including the IUCN Vulnerable bridled nailtail wallaby *Onychogalea fraenata* (Gould) [165,166], rock wallabies *Petrogale* spp. [159,164], and burrowing bettongs *Bettongia lesueur* (Quoy & Gaimard) [51].

Bandicoots and bilbies more commonly feature in fox and dingo diets than in cat diets, but occur twice as frequently in the diets of dingoes than foxes. For example, northern brown bandicoots (included on the dingo top food list) are present in 47.9% of dingo diet samples on Fraser Island [98] and 58% of samples from the Brisbane Valley [167]. At certain times, bilbies are present in 43% of dingo samples at Astrebla Downs National Park, where dingoes represent a significant threat to this remnant population [168]. Echidna was also on the dingo top food list, albeit with a low overall average FOO of 4 ± 4% across *n* = 73 studies/timepoints reporting their presence (FOO 27.9% [145], 10.2% [163], 10.8% [169], 13%, Fleming P.J.S., unpublished data). Platypus are seldom recorded ([170], Fleming P.J.S., unpublished data).

Several threatened vertebrate taxa were sufficiently commonly consumed to be distinctive to dingo diets. Bare-nosed wombats were on the dingo top food list, with one study reporting them present in 161 out of 314 scat samples (FOO 51% [171]) and others reporting wombat in more than 1 in 10 dingo diet samples (FOO 15% [90], 11% [172], 13% [170], 16% [173], 11% [151]). Dingo predation represents a significant threat to critically endangered northern hairy-nosed wombat *Lasiorhinus krefftii* (Owen) populations, one of the rarest land mammals in the world [174,175]. Koalas also appear in dingo diet studies (FOO 1.0% [167], 1.0% [166]) and mortality due to attacks by dingoes and free-ranging domestic dogs (43% of 18 mortalities for 39 koalas tracked for up to 3 years [176], 11 of 12 depredated koalas [177]) has been identified as a key factor affecting persistence of koala populations [178].

Finally, although fish are not often reported in any of the three predators' diets, fish were identified as distinctive of dingo diets. This result is likely weighted by the high incidence of fish in four separate studies on Fraser Island, Queensland (summarized by Behrendorff *et al*. [98]), where fish presence likely represents scavenging around human activities [179]. Other studies have a lower representation of fish in dingo diet.

### 4.4. Traits of prey taken

Previous studies have correlated habitat and physical or behavioural traits of Australian mammal species with their conservation status, identifying greater extinction and vulnerability for terrestrial species between 35 g–5.5 kg body mass, especially species from arid areas [11–14]. Traits of native species have also been analysed to investigate whether they can explain differential vulnerability to eutherian predators [39–41]. These traits included prey and predator species body mass, habitat preferences, foraging and anti-predator behaviour, diel activity patterns, refugia, mobility and fecundity. Here, we have quantified the presence of native mammal, bird and squamate prey (and FOO for mammal prey) in the diets of these three predators, which can further aid assessments of their predatory impacts on specific prey.

We show that prey body mass was the strongest trait associated with mammals, birds and squamates being recorded in dietary studies, with cats preferring small prey and dingoes preferring large prey. The cumulative likelihood of cat, fox and dingo predation (i.e. as recorded in diet studies) is most concentrated on intermediate-sized mammal prey. One caveat of these results is that we expect there to be many small squamate species that are preyed on by all three predators, particularly cats, but which have not been recorded in dietary studies (see secton below *Biases for diet results*). Less biased sampling of squamates may reveal the shape of the plotted curve for this relationship (figure 2) to be more like that of birds, with greater likelihood of predation for smaller species than currently predicted.

We found greater FOO of mammal species that use more open habitat in cat and fox diet studies, greater likelihood of birds that use open habitat in cat diet, and greater likelihood of squamates that use open habitat in fox and dingo diets. These findings support the hypothesis that loss of vegetation cover results in prey animals being more exposed to these predators (e.g. [131–133,180]). The recent 2019/2020 megafires in Australia, which resulted in an unprecedented area of temperate forest, woodland and shrubland across southeastern Australia being burnt [181], also highlight the role that climate change may play in further modifying habitat to favour cats and foxes (e.g. [133,182]). We also recorded a strong pattern of greater vulnerability for ground-foraging and ground-nesting bird species, which were more likely to appear in the diets of all three predator species. Presumably, this reflects increased chances of encountering these species and/or relative ease of capture.

### 4.5. Can spatial and temporal patterns in predator diets inform the question of mesopredator release?

Dietary studies can show the potential for interference competition if an intraguild predator occurs in the diet of another. There is some evidence of direct consumption of cats by dingoes. For example, several studies report FOO cat greater than 4% [91,130,161,168,183,184]. An experimental trial reported that a pair of dingoes killed at least three of six cats released into a fenced paddock with them [185]. Conversely, there is scant evidence that cats consume dingo, with only four studies reporting dingo in cat diet samples (single samples [58,130,184], 2 of 47 samples, believed to represent scavenging on culled dingoes [59]). Reports of consumption of cats by foxes in Australia are also uncommon [186–188]. While several studies report low incidence (FOO > 4%) of cat in fox diets, Woolley & Valente [189] reported cat in 12 of 66 fox scat samples from the Fitzgerald River National Park in Western Australia. Conversely, Leis [190] reported fox in 1 of 46 cat stomachs and 9 of 63 scat samples at Southern Downs, Queensland, the only study to report fox in cat diet samples. Few studies have reported fox presence in dingo diets (FOO > 4% [93,100]), although dingoes do kill foxes (e.g. [185]) and have also been observed to eat them (e.g. [93]). The low reporting of fox in dingo diets could reflect that foxes are rarely encountered—they are in lower densities where dingoes (and therefore dingo diet studies) are more common due to habitat preferences, predation or exclusion by humans (e.g. [39,191,192]) or simply because they are avoiding dingoes [193]. As well as potentially representing scavenging, traces of predator hair could indicate coprophagy for nutritional gain, as has been reported for foxes [194,195] and modern dogs [196,197].

Although only finding trace evidence does not discount direct physical attack, because predators may kill but not consume other (usually smaller) predators (e.g. [185]), the scant evidence of predators in diet samples supports the conclusion that intraguild predation is not common between dingoes, foxes and cats. Dietary studies therefore provide little support for mesopredator suppression via interference competition. Interference competition could also be manifest through altered behaviour of the mesopredators, affecting the timing of their activities. For example, there is increasing evidence that cats alter their activity patterns in the presence of dingoes (e.g. [198,199]), as they do around foxes (e.g. [200]) and Tasmanian devils (e.g. [201]). The mesopredators could also alter their spatial distribution [202] as suggested under the ‘landscape of fear’ hypothesis [203–205]. We suggest that stronger evidence for interference competition between dingoes, cats and foxes could come from experimental manipulations and behavioural interaction studies rather than dietary assessment.

Dietary competition is also a possible mechanism for mesopredator population suppression if there is overlap in resource use, particularly where this would limit survival, reproduction and therefore abundance of subordinate predators [206]. However, there is little evidence from the present study of dietary competition between cats and dingoes, with these two predators having quite distinctive diets. For example, cats are three times more likely to have eaten squamates and birds than dingoes, while dingoes are 12 times more likely to have consumed macropods, 29 times more likely to have consumed monotremes and 96 times more likely to have consumed wombats. Their diets are therefore distinctive enough to suggest that it would be unlikely for dingoes to directly compete with cats for their preferred food resources. If dingoes were to suppress cat numbers through exploitation competition, it may be more likely in arid areas, where there are fewer ecological niches (and therefore fewer potential prey taxa) and many prey items are also likely to be relatively scarce [207]; leading to the present finding that dietary overlap is greatest in arid areas for all three predators.

Cats are facultative diet specialists [208]—they switch diet when other profitable prey is available. For example, short-term experimental (approximately 80%) reduction in rabbits in a 37 km^2^ enclosure contributed to a 40% reduction in cat activity and survival of VHF-collared cats, which increased their consumption of squamates, birds and invertebrates [209]. Cats also switch between prey such as rabbits and breeding seabirds as they are seasonally available [55]. Significant shifts in predator diets have been demonstrated between boom and bust phases for predators in desert environments, where productivity is strongly linked with rainfall [210]. Under such scenarios, dietary overlap between the three predators is greatest during rodent irruptions [161,210,211], with foxes and dingoes substantially increasing their consumption of small mammals [210]. When the rodents return to more ‘normal’ numbers during bust periods, cat dietary breadth shows the least variation as they continue to primarily hunt small mammals, while foxes and dingoes increase their dietary range, with foxes switching to more vegetation and invertebrates, and dingoes switching to more squamates and large mammals [210]. It is probably therefore not surprising that, although we report a reduction in lagomorphs in cat diet over the last 55 years, a continuous supply of birds, reptiles and small mammals is likely to make up the staple diet of feral cats, with no significant long-term temporal change in overall mammal prey composition detecteds.

Foxes show broad dietary overlap with cats and are therefore potential food competitors. Over a three-year study at Lake Burrendong, New South Wales, after control efforts led to a reduction in fox abundance indices, cats showed a short-term increase in consumption of invertebrates and carrion, and were more likely to forage in open habitats [200]. Across an approximate 30-year comparison, however, removing foxes from Phillip Island did not significantly (*p* = 0.075) change the composition of cat diet [212,213]. Such long-term datasets are hard to standardize for potential seasonal effects (e.g. [102,212]), sampling locations (e.g. [212]) or demographic differences (e.g. [54]) that can influence interpretation of differences in diets, but nonetheless provide important insights over a long timeframe.

### 4.6. Biases for diet results

There are significant biases in the analysis of diet from scats compared with stomach contents due to differential digestibility of particular diet items (but see [214]). The identification of less plant [215,216], and the underreporting of amphibians [23,32] from scats has been identified previously. Bias towards reporting of mammal material from scats is also a common finding [215,216], while the soft tissues of large mammal species (e.g. livestock and other ungulates) are less likely to be reported from scats [23,217]. Therefore, sample type influences the detection and identification of prey remains, highlighting the importance of comparing like-for-like when contrasting predator diets. Although there has been suggestion of underreporting of birds in carnivore diet interpreted from scats analysis [215,216,218], this was not apparent in the present investigation by comparison between studies using different methods. Studies applying new metabarcoding molecular methods for diet analyses (e.g. [218–220]) are likely to detect scarce and readily digestible items from both stomach contents and scats, and will offer additional future insight.

In addition to differential digestibility issues, there has also been unreliable reporting of particular diet items, particularly for plant material, amphibians, soft-bodied invertebrates (e.g. earthworms [221]), and fish in the diets of these three predators. It would be beneficial for future studies to explicitly record true zeros for principal food groups that are absent.

Such biases could contribute to some of the apparent differences in diet composition identified in the present study. Despite a greater number of fox and dingo studies (and diet samples) included in this review, there were substantially fewer prey identified to species for foxes and dingoes compared with cats. There are several potential explanations for this finding.

(i) It may simply reflect the difficulty of identifying prey remains to species from scats (compared with stomach content analyses) given the marked differences in sample types for the three predators compared in this study, with only 48% of cat diet studies using scats, compared with 65% of fox and 88% of dingo diet studies.
(ii) There may also be differences in the degree to which these predators masticate their prey before swallowing them. Small prey (such as many of the small squamate species consumed by cats) are more likely to be swallowed whole, while larger prey species tend to be chewed more and are therefore less identifiable.
(iii) Cats consume more species of small prey than either foxes or dingoes, and there are more species of small than large squamates, birds and mammals.
(iv) Cats also eat greater numbers of smaller prey, increasing the likelihood of detecting multiple species/animals in their diet.

In addition to such spatial and temporal biases in diet surveys, biases due to sampling method therefore also need to be acknowledged, emphasizing the need to compare like-for-like when making assessment of dietary differences; to account for this potential bias, we included sample type as a variable in our diet composition analyses. Furthermore, interpretation of predation threat from diet data is particularly challenging for range-restricted prey species that can have reduced likelihood of appearing in predator diets simply due to limited sampling carried out within their geographical range. For example, there may be a large number of small squamate species in Australia that are eaten by cats but which have not been detected because dietary studies have not overlapped the reptile's small geographical range [222,223].

There are also problems for scarce species that will only rarely (if at all) appear in diet samples [224]. For example, feral cats and foxes are believed to threaten the ground-foraging and -nesting western ground parrot (*Pezoporus flaviventris*) [225], but there has been no evidence of this species in the predators' diet [226]. Similarly, foxes and cats are recognized as a key threat for the numbat (*Myrmecobius fasciatus*) [227]. Numbats declined upon the arrival of foxes into their geographical range, predator control under the Western Shield fox control programme has contributed to an increase in numbat population, and cat predation has been responsible for the killing of translocated animals [227]. Despite these damning data, there has been no recorded evidence of numbats in either fox or cat diets. However, every animal killed can reduce the population or prevent recruitment of young, and the absence of evidence in predator diet studies can therefore be misleading with regard to predation risk.

## 5. Conclusion

This study, based on a massive dataset spanning *ca.* 70 years and at a continental scale, demonstrates substantial overlap but also important differences among the diets of three predator species now occurring across most of Australia. Many previous studies demonstrate substantial conservation impacts of this predation. Dietary studies, such as that reported here, can provide clues regarding the impact of predators on threatened species populations, although such evidence needs to be considered in light of limitations of the method of study. Molecular methods may prove more effective in detecting scarce species from scats [220] in order to elucidate predation risk for such species.

Prey consumed reflects differences in predator body mass and the hunting and feeding strategies of the feral cat, fox and dingo. Cats and dingoes consume preferred prey (smaller and larger prey respectively) when these are available, while foxes are more generalist in their dietary habits. Dietary studies can therefore be used to build our understanding of potential competition between these predators, and therefore the likelihood that dingoes could effectively suppress cats and foxes. Cats and dingoes have the least dietary overlap of the three predators, especially for more mesic sites, suggesting dietary competition with dingoes is highly unlikely to suppress cat numbers. Foxes show substantial dietary overlap with both cats and dingoes, but their diverse and opportunistic diet suggests that foxes will simply switch food sources to mitigate competition for food resources with dingoes. Neither the cat nor fox is therefore likely to be greatly influenced by dietary competition with dingoes.

Finally, our review captures evidence that nearly half (45%) of all Australian terrestrial mammal, bird and squamate species are known to be consumed by at least one of these three predator species. The introduced fox and cat have had dramatic recorded impact on the Australian fauna, contributing to the catastrophic loss of biodiversity across the continent [5,14]. However, dingo predation also threatens some native species (e.g. [51,89,165,174,175,177]). As it does for introduction of carnivores anywhere in the world, proposed ‘rewilding’ programmes—re-introducing dingoes for conservation benefit [20,228]—need balanced consideration of both benefits and potential costs of their re-introduction for each specific location.

## Data Availability

This study is a review of previously published and some unpublished diet studies (raw data available at doi:10.5061/dryad.612jm646j [229]). The calcualted data are provided in electronic supplementary material [230].
